# ﻿Unveiling species diversity within Mortierellomycota from China X: Three new species in *Linnemannia* and one in *Mortierella*

**DOI:** 10.3897/mycokeys.125.168474

**Published:** 2025-11-20

**Authors:** Xin-Yu Ji, Zi-Ying Ding, Wen-Xiu Liu, Fei Li, Heng Zhao, Shi Wang, Xiao-Yong Liu

**Affiliations:** 1 College of Life Sciences, Shandong Normal University, Jinan 250358, China Shandong Normal University Jinan China; 2 CAS Key Laboratory of Forest Ecology and Silviculture, Institute of Applied Ecology, Chinese Academy of Sciences, Shenyang 110016, China Institute of Applied Ecology, Chinese Academy of Sciences Shenyang China; 3 Institute of Microbiology, Chinese Academy of Sciences, Beijing 100101, China Institute of Microbiology, Chinese Academy of Sciences Beijing China

**Keywords:** Mortierellaceae, Mucoromycota, multi-gene phylogeny, taxonomy, Zygomycota

## Abstract

The species of the Mortierellaceae family are diverse and widely distributed. Four new species in this family are proposed from rhizosphere soil through a comprehensive taxonomic approach that combined multi-locus (SSU-ITS-LSU-*RPB1*-*Act*) phylogenetic analyses with detailed morphological examination. This study describes and illustrates these taxa, clarifying their morphological features from closely related species and their phylogenetic positions within the family. *Linnemannia chlamydospora***sp. nov.** (phylogenetically proximate to *L. longigemmata*) is characterized by the abundant production of thick-walled chlamydospores. *Linnemannia ovalispora***sp. nov.** (a sister taxon to *L. rugosa*) is distinguished by its oval sporangiospores. *Linnemannia yunnanensis***sp. nov.** (closely allied to *L. bainierella*) is characterized by its oval chlamydospores and is named after Yunnan Province, its type locality. *Mortierella irregularispora***sp. nov.** (clustering with *M. parvispora*) is distinguished by its irregularly shaped sporangiospores. As this is the tenth instalment of our systematic survey of Mortierellomycota diversity in China, this study expands the global species inventory of Mortierellaceae to 158.

## ﻿Introduction

Mortierellaceae species have an important industrial and ecological value. Species within the family Mortierellaceae are notable for their ability to synthesize polyunsaturated fatty acids (PUFAs), such as arachidonic acid (ARA), which are valuable for biofuel production and widely utilized in commercial sectors (Holland 2001; Yadav et al. 2014; Telagathoti et al. 2022). Some species can also synthesize enzymes (lipase, cellulase) with potential applications in biofuels and food processing (Holland 2001; Wagner et al. 2013; Yadav et al. 2014; Telagathoti et al. 2022). Many Mortierellaceae species also produce bioactive antimicrobial metabolites that serve as effective biocontrol agents against plant pathogens (Shemshura et al. 2018). Some Mortierellaceae species can participate in organic matter mineralization as decomposers, promote soil nutrient cycling, and improve plant stress resistance (Holland 2001; Wagner et al. 2013; Yadav et al. 2014; Shemshura et al. 2018; Telagathoti et al. 2022). Additionally, certain members of this family exhibit unique ecological and biotechnological capabilities, including plant growth promotion, restructuring of rhizosphere bacterial communities, and decomposition of organic litter, highlighting their multifaceted roles in both industrial and environmental contexts (Li et al. 2020; Ozimek and Hanaka 2021).

Mortierellaceae belongs to Mortierellomycota, Mortierellomycotina, Mortierellomycetes, and Mortierellales (http://www.indexfungorum.org/, accessed on 14 May 2025) (Smith et al. 2013; Wijayawardene et al. 2024). Mortierellaceae species can usually be isolated from soil, plant rhizomes, animal remains, and mosses (Tedersoo et al. 2014). The typical characteristics of Mortierellaceae species include white colony color, rosette-shaped colony morphology, and a distinctive odor generally described as resembling garlic or wet dog hair (Linnemann 1941; Gams 1977; Petkovits et al. 2011). This taxon is widely distributed throughout the country (Linnemann 1941; Holland 2001; Wagner et al. 2013; Yadav et al. 2014; Shemshura et al. 2018; Li et al. 2020; Ozimek and Hanaka 2021; Telagathoti et al. 2022). GBIF database documents Mortierellaceae from Africa (22,727 records, 5.59%), Antarctica (2,889, 0.71%), Asia (42,876, 10.55%), Oceania (47,301, 11.64%), Europe (231,446, 56.97%), North America (26,872, 6.61%) and South America (32,155, 7.91%; https://www.gbif.org/, accessed on 22 May 2025). In summary, the species of the family Mortierellaceae are mainly concentrated in Europe.

In recent years, Mortierellaceae has seen the discovery of many new species of *Mortierella* and *Linnemannia* (Holland 2001; Wagner et al. 2013; Yadav et al. 2014; Telagathoti et al. 2022), but other new genera remain to be further studied. The Catalogue of Life database contains 17 genera with a total of 144 species. Of these, *Mortierella* contains the largest number of species, 80, followed by *Linnemannia* with 24, then *Podia* and *Entomortierella* in equal third place, both with nine (https://www.catalogueoflife.org/, accessed on 22 May 2025).

In this study, extensive field sampling in Yunnan and Xizang, combined with detailed laboratory analyses, resulted in the discovery of three new species in *Linnemannia* and one in *Mortierella*. The newly described species are *Linnemannia chlamydospora*, *L. ovalispora*, *L. yunnanensis*, and *Mortierella irregularispora*. These species were identified based on molecular phylogenetic evidence, morphological characteristics, and growth temperature profiles. This is the tenth report in a series of studies on the diversity of Mortierellomycota across China (Tao et al. 2024; Wang et al. 2024; Zhao et al. 2024; Ding et al. 2025a; Ding et al. 2025b; Ji et al. 2025a; Ji et al. 2025b; Wang et al. 2025). These findings not only enrich the species diversity of the family Mortierellaceae but also provide new directions for the taxonomic and evolutionary study of the family.

## ﻿Materials and methods

### ﻿Isolation

Soil samples were collected from Yunnan and Xizang in 2024 following the protocols established by Zou et al. (2022) and Liu et al. (2019). Each soil sample (approximately 100 g) was transferred to sterile polyethylene bags labeled with collection date, vegetation type, elevation, and GPS coordinates (latitude/longitude) (Rathnayaka et al. 2025). All samples were stored at 4 °C post-transportation until laboratory processing. Pure strains were isolated from the soil samples using a combination of soil dilution plating and moist-chamber cultivation methods (Zhao et al. 2021). Soil suspensions were prepared by homogenizing approximately 1 g of soil sample in 10 mL of sterile deionized water within a 15 mL conical tube, followed by mechanical agitation on a vortex mixer for 25 minutes at 1,500 rpm to achieve thorough dispersion. Serial dilutions were performed by transferring 1 mL of the primary suspension into 9 mL of sterile deionized water, generating a 10^−2^ dilution. This process was repeated sequentially to obtain 10^−3^ and 10^−4^ dilutions. For fungal isolation, 200 μL aliquots of the 10^−3^ and 10^−4^ dilutions were aseptically pipetted onto Rose Bengal Chloramphenicol (RBC) agar plates. The medium contained per liter: peptone (5.00 g), KH_2_PO_4_ (1.00 g), MgSO_4_·7H_2_O (0.50 g), Rose Bengal dye (0.05 g), glucose (10.00 g), chloramphenicol (0.10 g), and agar (15.00 g), adjusted to pH 6.8 ± 0.2 (Corry et al. 1995). Samples were evenly distributed using flame-sterilized glass spreaders and incubated at 26 °C under light-restricted conditions for 2–5 days to promote fungal colony development. Subsequently, fungal colonies exhibiting active hyphal growth margins were selectively sub-cultured onto fresh Potato Dextrose Agar (PDA: 20 g/L glucose, 200 g/L potato infusion, 20 g/L agar, pH 5.6 ± 0.2) or malt extract agar (MEA: 33.6 g/L malt extract, 20 g/L agar) using inoculation needles. Macromorphological features were documented with a high-resolution digital imaging system (Canon PowerShot G7X, Canon, Tokyo, Japan). For the wet-chamber protocol, homogenized soil aliquots (1 g) were aseptically spread on PDA plates, sealed, and inverted incubated at 15 °C (±0.5 °C) in the dark to simulate the subsurface niche. After 48–72 h of incubation, isolate primary fungal colonies by quadrant streak using flame-sterilized inoculation loops. After two days, the agar containing mycelia at the edge of the colony was transferred to fresh PDA or MEA, and culture as described above. After about five days, the strain grew well.

### ﻿Morphological observation

Lactophenol cotton blue (LPCB) staining droplets were added to the glass slide. Then, a small piece of tape was touched to the mycelial surface, causing some hyphae to adhere to it. The stained specimen was immersed in a lactol cotton blue (LCB) solution for easy observation morphological analysis utilized a stereoscope (Olympus SZX10, Olympus, Tokyo, Japan) and a light microscope (Olympus BX53, OLYMPUS, Tokyo, Japan), and a high-definition color digital camera (Olympus DP80 OLYMPU, Tokyo, Japan) to observe hyphal structures and reproductive organs (Jiang et al. 2024; Tao et al. 2024; Wang et al. 2024; Ding et al. 2025a; Ding et al. 2025b; Ji et al. 2025a; Ji et al. 2025b; Wang et al. 2025). Morphometry analysis was performed using Digimizer software (v5.6.0) with at least 15 individuals measured per morphological trait. To determine the minimum and maximum growth temperatures of the strains, the temperature gradient method was used. First, initiate a thermal acclimatization protocol by incubating the primary culture at 10 °C (±0.5 °C) for 48 h to stabilize fungal metabolism. Subsequently, the incubation temperature was reduced by 1 °C increments per day until radial growth stopped. The final temperature before growth arrest is designated as the minimum growth temperature. All strains were stored in 10% sterile glycerol at -20 °C. The living cultures were stored in the China Microbiological Culture Collection Center, Beijing, China (CGMCC). Equivalent strains were preserved in the Shandong Normal University Culture Collection (XG). Dry cultures of types were submitted to the Herbarium Mycologicum Academiae Sinicae, Beijing, China (Fungarium; HMAS). The taxonomic information was deposited to the Fungal Names repository (https://nmdc.cn/fungalnames/).

### ﻿DNA extraction, PCR amplification, and sequencing

Genomic DNAs were extracted using the DNA Extraction Kit (Cat. No.: 70409-20; Beaver Biomedical Engineering Co., Ltd.) (Doyle et al. 1990; Wang et al. 2023). Target regions (ITS, LSU, SSU, *RPB1*, and *Act*) were amplified through PCR with primer pairs and protocols outlined in Table 1. Reactions were performed in a 25 μL final volume containing 12.5 μL of 2 × Hieff Canace Plus PCR Master Mix with dye (Yeasen Biotechnology, Cat No. 10154ES03), 9.5 μL of ddH_2_O, 1 μL of forward primer (10 μM), 1 μL of reverse primer (10 μM), and 1 µL of template genomic DNA (1 ng/μL). Amplified products were visualized on a 2% agarose gel at 254 nm and purified using the Gel Extraction Kit (Cat# AC0101-C; Shandong Sparkjade Biotechnology Co., Ltd.), designed for efficient recovery of DNA fragments ranging from 100 bp to 20 kb (Zhang et al. 2022; Zhang et al. 2025). The same supplier also provided the RNA Rapid Extraction Kit (Cat# AC0305; Shandong Sparkjade Biotechnology Co., Ltd.), which was available for total RNA extraction had transcriptomic analyses been required. DNA sequencing was performed by Beijing Tsingke Biotech Co., Ltd. All sequences generated in this study were deposited in GenBank (accession numbers provided in the Suppl. material 1), in accordance with standardized submission protocols for public data accessibility.

**Table 1.** PCR information used in this study.

Locus	PCR primers	Primer sequences (5’–3’)	PCR cycle	Reference
ITS	ITS5 ITS4	GGA AGT AAA AGT CGT AAC AAG G TCC TCC GCT TAT TGA TAT GC	95 °C 5 min; (95 °C 30 s, 55 °C 30 s, 72 °C 1 min) × 35 cycles; 72 °C 10 min	(White et al. 1990)
LSU	LR0R LR5	GTA CCC GCT GAA CTT AAG C TCC TGA GGG AAA CTT CG	95 °C 5 min; (94 °C 30 s, 52 °C 45 s, 72 °C 90 s) × 30 cycles; 72 °C 10 min	(Hurdeal et al. 2023)
SSU	NS1 NS4	GTA GTC ATA TGC TTG TCT CC CTT CCG TCA ATT CCT TTA AG	95 °C 5 min; (94 °C 60 s, 54 °C 50 s, 72 °C 60 s) × 37 cycles; 72 °C 10 min	(Hurdeal et al. 2023)
RPB1	RPB1-Af RPB1-Cr	GAR TGY CCD GGD CAY TTY GG CCN GCD ATN TCR TTR TCC ATR TA	95 °C 3 min; (94 °C: 40 s, 60 °C: 40 s, 72 °C: 2 min) × 9 (94 °C: 45 s, 55 °C: 1.5 min, 72 °C: 2 min) × 37 cycles; 72 °C 10 min	(Stiller and Hall 1997)
Act	ACT-1 ACT-4R	TGG GAC GAT ATG GAI AAI ATC TGG CA TC ITC GTA TIC TIG CTI IGA IAT CCA CA T	95 °C 3 min; (95 °C: 60 s, 55 °C: 60 s, 72 °C: 1 min) × 30 cycles; 72 °C 10 min	(Voigt and Wöstemeyer 2000)

### ﻿Phylogenetic analyses

The newly acquired sequence data were processed using MEGA v7 to ensure consistency (Kumar et al. 2016; Larsson 2014). Reference sequences for Mortierellaceae were retrieved from GenBank using the methodology of Telagathoti et al., and phylogenetic analyses were conducted for each genetic marker (Telagathoti et al. 2022). The evolutionary relationships within Mortierellaceae were reconstructed through both Maximum Likelihood (ML) and Bayesian Inference (BI) approaches, implemented via the CIPRES Science Gateway (https://www.phylo.org/, accessed 16 May 2025) (Nie et al. 2020a; Nie et al. 2020b). The maximum likelihood (ML) analysis was performed using RAxML version 8.2.4 on the CIPRES Science Gateway Version 3.3 platform, with 1,000 bootstrap replicates conducted to assess the robustness of the phylogenetic tree (Miller et al. 2010; Nguyen et al. 2015). The Bayesian inference (BI) analysis was conducted using the GTR + I + G model, with samples collected every 1,000 generations. A total of eight cold Markov chains were run concurrently for two million generations (Ronquist et al. 2012; Stamatakis 2014). The resulting phylogenetic trees were visually optimized and annotated using iTOL (https://itol.embl.de, accessed May 16, 2025) and Adobe Illustrator CC 2019 (Wang et al. 2023).

## ﻿Results

### ﻿Phylogeny

**Figure 1.**
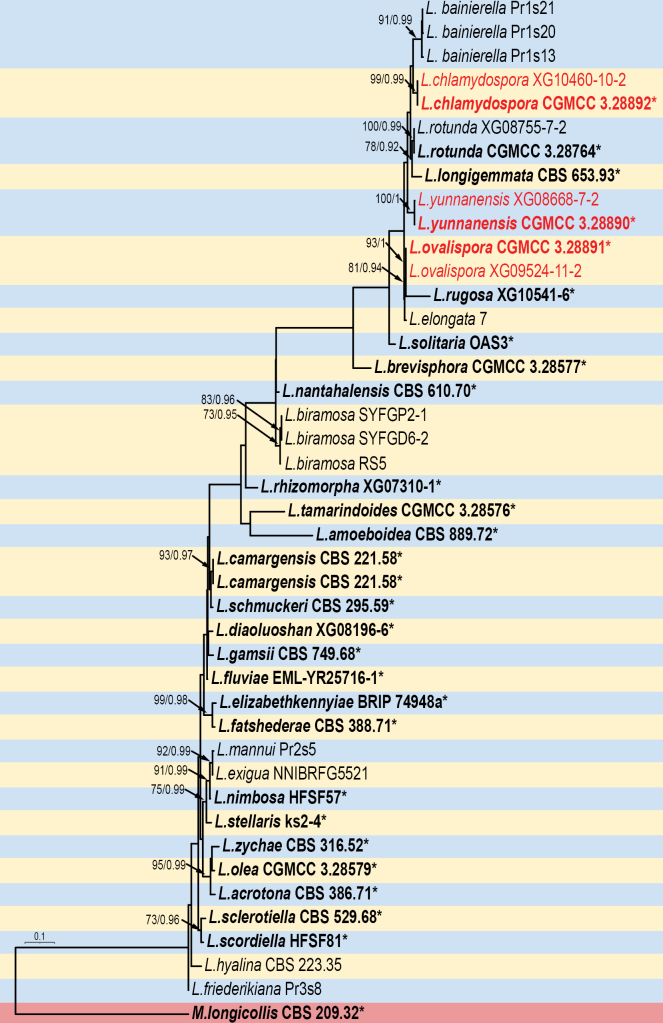
The Maximum Likelihood phylogenetic tree for the genus *Linnemannia* based on combined ITS, LSU, SSU, *RPB1*, and *Act* sequences with *Mortierella longicollis* as outgroup. Node supports are indicated by two metrics: Maximum Likelihood Bootstrap Values (left, MLBV ≥ 70%) and Bayesian Inference Posterior Probabilities (right, BIPP ≥ 0.90), separated by a slash (/). Novel species are emphasized in red. Bold entries with asterisks (*) denote ex-type or ex-holotype strains. The scale bar in the lower left represents 0.1 substitutions per site.

For *Linnemannia*, phylogenetic analyses were performed on a dataset comprising 43 strains representing 34 species, with *Mortierella longicollis* (CBS 879.97) as an outgroup (Genbank numbers see Suppl. material 1: table S1). The sequence matrix comprises 4,863 concatenated characters: 1–690 (ITS), 691–1,672 (LSU), 1,673–2,728 (SSU), 2,729–4,099 (*RPB1*), and 4,100–4,863 (*Act*). Among these characters, 1,130 are parsimony-informative, along with 3,417 constant and 316 parsimony-uninformative. Bayesian tree topology is congruent with that of the ML tree (Fig. 1).

**Figure 2.**
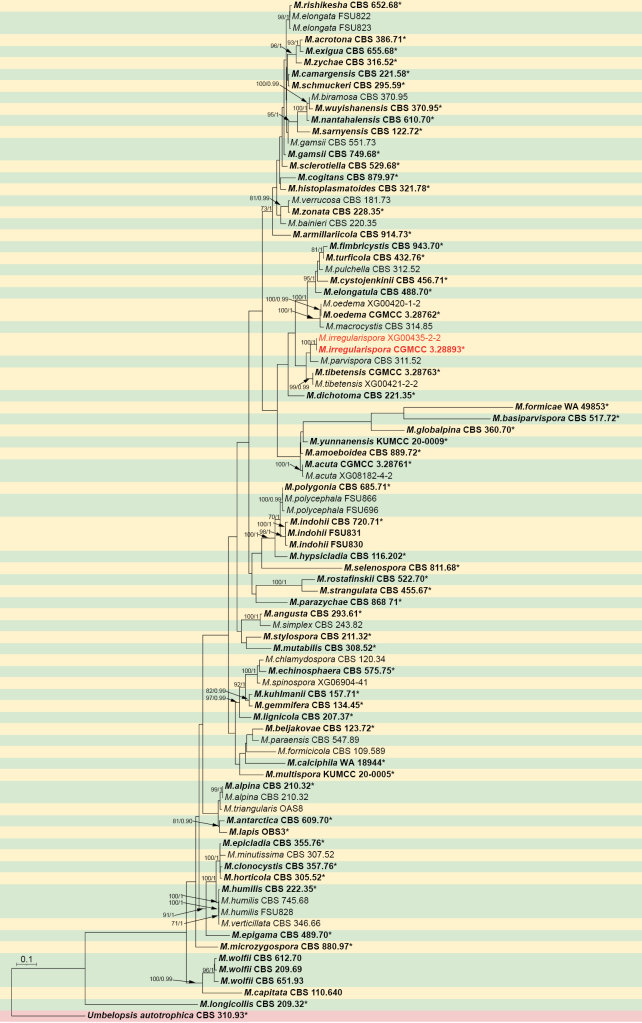
The Maximum Likelihood phylogenetic tree for the genus *Mortierella* based on combined ITS, LSU, SSU, *RPB1*, and *Act* sequences with *Umbelopsis autotrophica* as an outgroup. Node supports are indicated by two metrics: Maximum Likelihood Bootstrap Values (left, MLBV ≥ 70%) and Bayesian Inference Posterior Probabilities (right, BIPP ≥ 0.90), separated by a slash (/). Novel species are emphasized in red. Bold entries with asterisks (*) denote ex-type or ex-holotype strains. The scale bar in the lower left represents 0.1 substitutions per site.

For *Mortierella*, phylogenetic analyses were performed on a dataset comprising 89 strains representing 75 species, with *Umbelopsis autotrophica* (CBS 310.93) as an outgroup (Genbank numbers see Suppl. material 1: table S2). The sequence matrix comprises 5,292 concatenated characters: 1–962 (ITS), 963–1,968 (LSU), 1,969–3,049 (SSU), 3,050–4,422 (*RPB1*), and 4,423–5,292 (*Act*). Among these, 1,806 are parsimony-informative, along with 2,581 constant and 905 parsimony-uninformative. Bayesian tree topology is consistent with the ML tree (Fig. 2).

### ﻿Taxonomy

#### ﻿ Linnemannia chlamydospora

Taxon classificationFungiMortierellalesMortierellaceae

X.Y. Ji, H. Zhao & X.Y. Liu sp. nov.

3C17D53B-DE95-5D72-B3FB-DDC999B60972

Fungal Names: FN 572057

Fig. 3

##### Type.

China • Xizang, Nyingchi City, Bayi District (29°33'40"N, 94°33'13"E, altitude 3713 m), from soil, 29 August 2024, X.Y. Ji, holotype HMAS 354073, ex-holotype living culture CGMCC 3.28892 (=XG10460-10-1).

##### Etymology.

The epithet *chlamydospora* (Lat.) refers to a larger number of chlamydospores.

##### Description.

Colonies on PDA at 16 °C for 5 d, reaching 70 mm diameter, fast growing with a rate of 14 mm/d, garlic smell, annual ring-like. Hyphae hyaline, 2.0–7.8 µm wide (n = 15, x̄ = 4.8). Sporangia mostly spherical, smooth, hyaline, 9.0–12.7 µm in diameter (n = 15, x̄ = 11.2). Sporangiospores hyaline, smooth, mostly round, 5.8–12.7 µm in diameter (n = 15, x̄ = 9.6). Chlamydospores abundant, oval, round, and irregular, 11.2–33.6 µm long and 6.8–21.7 µm wide (n = 15, x̄ =17.4 × 13.0 µm). Zygospores not found.

**Figure 3.**
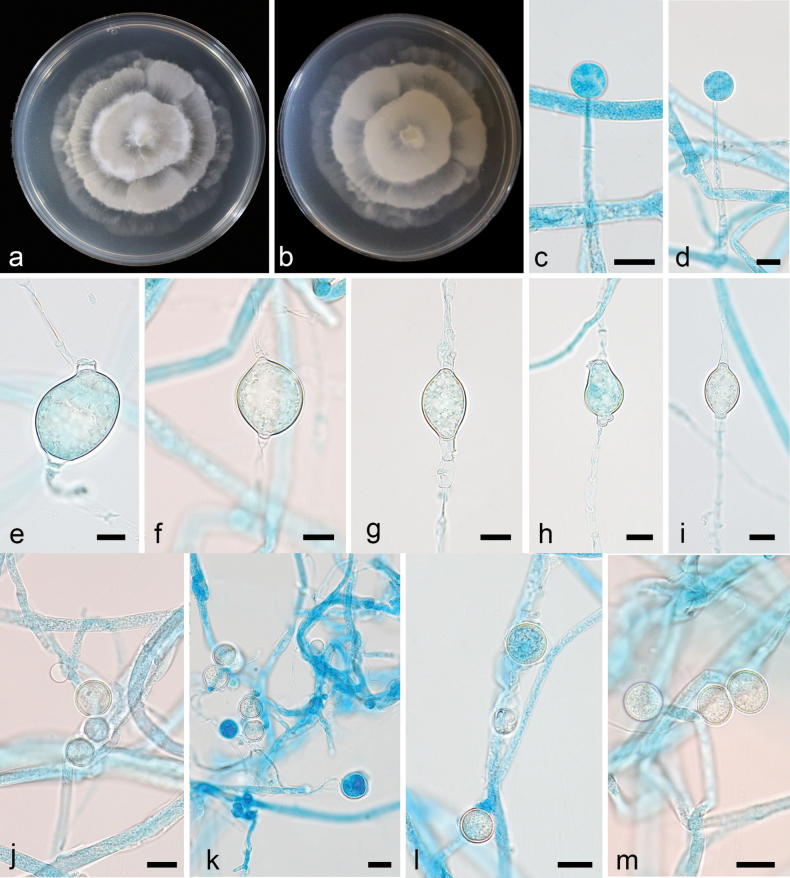
*Linnemannia chlamydospora* ex-holotype CGMCC 3.28892. **a, b.** Colonies on PDA (**a.** obverse; **b.** reverse); **c, d.** Sporangia; **e–i.** Chlamydospores; **j–m.** Sporangiospores. Scale bars: 10 µm (**c–m**).

##### Temperature requirements.

Minimum growth temperature 4 °C and maximum growth temperature 28 °C.

##### Additional strains examined.

China • Xizang, Nyingchi City, Bayi District (29°33'40"N, 94°33'13"E, altitude 3713 m), from soil, 29 August 2024, X.Y. Ji, living culture XG10460-10-2.

##### Notes.

The phylogenetic analysis showed that the new species *L. chlamydospora* is closely related to *L. longigemmata* (Fig. 2). It is distinguished from *L. longigemmata* by 55/621 characters in ITS sequences. Morphologically, compared to *L. longigemmata*, the new species has larger sporangiospores (5.8–12.7 µm vs 5.0–9.0 µm) and smaller chlamydospores (11.2–33.6 × 6.8–21.7 µm vs up to 60.0 µm).

#### ﻿ Linnemannia ovalispora

Taxon classificationFungiMortierellalesMortierellaceae

X.Y. Ji, H. Zhao & X.Y. Liu sp. nov.

D0958ECE-D54C-5126-B6B9-F19A157DB04E

Fungal Names: FN 572920

Fig. 4

##### Type.

China • Yunnan Province, Yuxi City, Research and service area of Hongta District (24°15'41"N, 102°28'58"E, altitude 1632.18 m), from soil, 15 March 2024, X.Y. Ji, holotype HMAS 354072, ex-holotype living culture CGMCC 3.28891 (=XG09524-11-1).

##### Etymology.

The epithet *ovalispora* (Lat.) refers to the oval sporangiospores.

##### Description.

Colonies on MEA at 16 °C for 5 d, attaining 60 mm diameter, moderately fast growing with a rate of 12 mm/d, garlic smell, with a thin white film and few aerial mycelia. Hyphae hyaline, 1.4–4.5 µm in diameter (n = 15, x̄ = 2.4). Sporangiospores transparent, mostly oval, 3.8–9.8 × 2.7–6.2 µm (n = 15, x̄ = 6.7 × 4.3 µm). Chlamydospores present, gastric-shaped, 7.7–9.2 × 5.7–8.8 µm (n = 15, x̄ = 8.4 × 7.3 µm). Zygospores absent.

**Figure 4.**
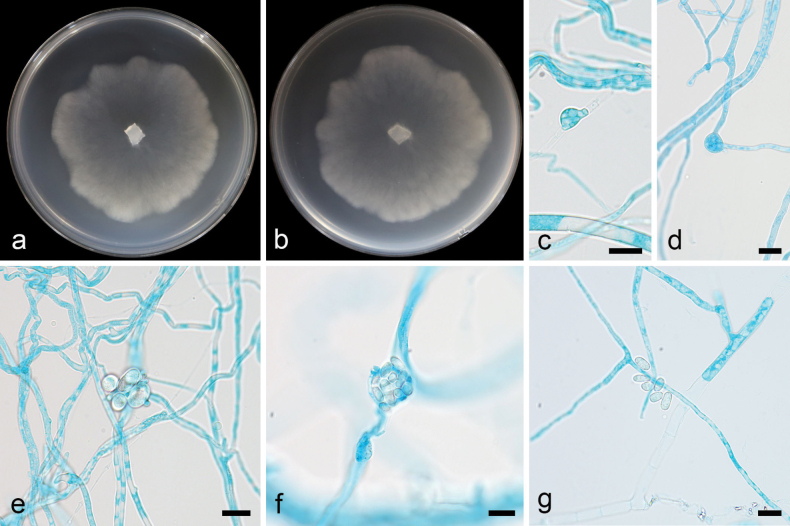
*Linnemannia ovalispora* ex-holotype CGMCC 3.28891. **a, b.** Colonies on MEA (**a.** obverse; **b.** reverse); **c, d.** Chlamydospores; **e–g.** Sporangiospores. Scale bars: 10 µm (**c–g**).

##### Temperature requirements.

Minimum growth temperature 4 °C and maximum growth temperature 29 °C.

##### Additional strains examined.

China • Yunnan Province, Yuxi City, Research and service area of Hongta District (24°15'41"N, 102°28'58"E, altitude 1632.18 m), from soil, 15 March 2024, X.Y. Ji, living culture XG09524-11-2.

##### Notes.

Integrated phylogenetic analysis of the ITS, LSU, SSU, *RPB1*, and *Act* gene regions revealed that the new species *Linnemannia ovalispora* is closely related to *L. rugosa* (Fig. 2). The new species is distinguished from *L. rugosa* by 82/630 and 80/1059 characters in ITS and LSU sequences, respectively. In terms of sporangiospores, the new species is oval and smooth, unlike *L. rugosa*, which is round and coarse. In the shape of chlamydospores, the new species is gastric-shaped, while *L. rugosa* is elongated.

#### ﻿ Linnemannia yunnanensis

Taxon classificationFungiMortierellalesMortierellaceae

X.Y. Ji, H. Zhao & X.Y. Liu sp. nov.

0A94A126-42F3-5BAF-B158-C8FFE7D15C59

Fungal Names: FN 572921

Fig. 5

##### Type.

China • Yunnan Province, Yuxi City, Xinping Dai Autonomous County, Ancient Tea Horse Road (23°57'28"N, 101°30'38"E, altitude 2196.56 m), from soil, 16 May 2024, X.Y. Ji, holotype HMAS 354071, ex-holotype living culture CGMCC 3.28890 (=XG08668-7-1).

##### Etymology.

The epithet “*yunnanensis*” (Lat.) refers to the type location, Yunnan province.

##### Description.

Colonies on PDA at 16 °C for 5 d, reaching 57 mm diameter, moderately fast growing with a rate of 11.4 mm/d, garlic smell, with sparse aerial mycelia, rose patterned. Hyphae hyaline, 2.5–7.4 µm in diameter (n = 15, x̄ = 5.2), sometimes swollen. Sporangia deliquescent-walled. Sporangiospores smooth, hyaline, mostly round, 5.0–9.8 µm in diameter (n = 20, x̄ = 7.3 µm). Chlamydospores present, mostly oval, 13.3–18.1 × 7.3–15.0 µm (n = 15, x̄ = 16.5 × 9.8 µm). Zygospores not found.

**Figure 5.**
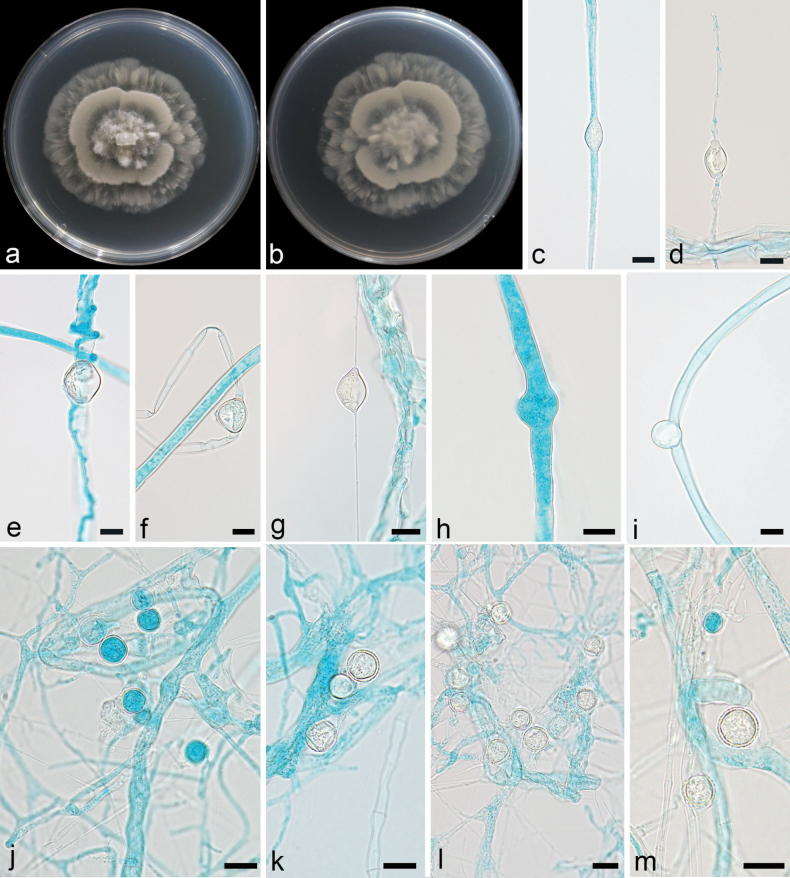
*Linnemannia yunnanensis* ex-holotype CGMCC 3.28890. **a, b.** Colonies on PDA (**a.** obverse; **b.** reverse); **c–g.** Chlamydospores; **h, i.** Typical swollen hyphae; **j–m.** Sporangiospores. Scale bars: 10 µm (**c–m**).

##### Temperature requirements.

Minimum growth temperature 4 °C and maximum growth temperature 29 °C.

##### Additional strains examined.

China • Yunnan Province, Yuxi City, Xinping Dai Autonomous County (23°56'39"N, 101°30'1"E, altitude 2397.53 m), from soil, 16 May 2024, X.Y. Ji, living culture XG08668-7-2.

##### Notes.

Phylogenetic analysis of the five combined genes of ITS, LSU, SSU, *RPB1*, and *Act* showed that the new species *Linnemannia yunnanensis* is closely related to *L. bainierella* (Fig. 1). The new species is distinguished from *L. bainierella* by 141/640 and 137/1007 characters in ITS and LSU sequences, respectively. Morphologically, compared to *L. bainierella*, the new species has larger sporangiospores (5.0–9.8 µm vs 5.0–7.0 × 2.5–4.0 µm).

#### ﻿ Mortierella irregularispora

Taxon classificationFungiMortierellalesMortierellaceae

X.Y. Ji, H. Zhao & X.Y. Liu sp. nov.

266C963B-B9DE-5276-9F8B-065460F8556C

Fungal Names: FN 572922

Fig. 6

##### Type.

China • Xizang, Shigatse City, Nyalam County, (28°17'53"N, 85°58'26"E, altitude 4300 m), from soil, 27 June 2024, X.Y. Ji, holotype HMAS 354074, ex-holotype living culture CGMCC 3.28893 (=XG00435-2-1).

##### Etymology.

The epithet *irregularispora* (Lat.) refers to irregular sporangiospores.

##### Description.

Colonies on PDA at 16 °C for 5 d, reaching 53 mm diameter, slow growing with a rate of 10.6 mm/d, with a wet dog smell, with sparse aerial mycelia. Hyphae hyaline, upright or bent, sometimes swollen. Sporangiophores arising from aerial mycelia, erect or slightly bent, unbranched, 48.6–176.7 µm long (n = 15, x̄ = 89.5 µm), 4.3–7.3 µm (n = 15, x̄ = 5.3 µm) wide at the base, and 2.2–3.0 µm (n = 15, x̄ = 2.8 µm) wide at the top. Sporangia almost spherical, smooth, 7.3–19.6 µm in diameter (n = 15, x̄ = 15.0). Sporangiospores hyaline, with irregular shape, smooth, 3.3–4.7 µm vertical height (n = 15, x̄ = 4.0). Chlamydospores absent. Zygospores not observed.

**Figure 6.**
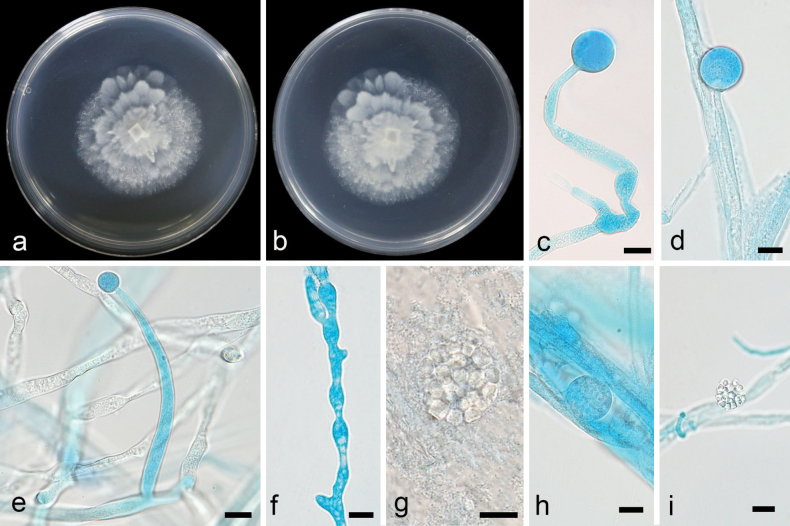
*Mortierella irregularispora* ex-holotype CGMCC 3.28893. **a, b.** Colonies on PDA (**a.** obverse; **b.** reverse); **c–e, h.** Sporangia; **f.** Swollen hyphae; **g, i.** Sporangiospores. Scale bars: 10 µm (**c–i**).

##### Temperature requirements.

Minimum growth temperature 4 °C, and maximum growth temperature 28 °C.

##### Additional strains examined.

China • Xizang, Shigatse City, Nyalam County (28°17'53"N, 85°58'26"E, altitude 4300 m), from soil, 26 June 2024, X.Y. Ji, living culture XG00435-2-2.

##### Notes.

Phylogenetic analysis of five loci (ITS, LSU, SSU, *RPB1*, and *Act*) showed that the new species *M. irregularispora* is closely related to *M. parvispora* (Fig. 2). The new species is distinguished from *M. parvispora* by 46/629, 117/1061, and 27/1019 characters in ITS, LSU, and SSU sequences, respectively. Morphologically, compared to *M. parvispora*, the new species has smaller sporangia (7.3–19.6 µm vs 20.0–35.0 µm), larger sporangiospores (3.3–4.7 µm vs 2.0–3.0 µm), and no chlamydospores.

## ﻿Discussion

In this study, morphological and molecular analyses of Mortierellaceae species confirmed the existence of four new species: *Linnemannia chlamydospora*, *L. ovalispora*, *L. yunnanensis*, and *Mortierella irregularispora*. Analysis of these data provided strong support for the clades of these species. Phylogenetic placement of the isolates was strongly supported: *L. yunnanensis* achieved 100% MLBV and 1.00 BIPP; *L. chlamydospora*, 99% MLBV and 0.99 BIPP; *L. ovalispora*, 93% MLBV and 1.00 BIPP; and *M. irregularispora*, 100% MLBV and 1.00 BIPP (see Fig. 1 and Fig. 2). Phylogenetic trees based on five loci revealed the genetic relationship between these four new species and their neighbors. In the phylogenetic tree, it can be observed that all four new species are closely related to themselves, and we find differences between them in terms of molecules and morphology (Chen 1992; Telagathoti et al. 2022). *Linnemannia chlamydospora* and *L. longigemmata* are closely related. Compared to *L. longigemmata*, *L. chlamydospora* has larger sporangiospores and smaller chlamydospores. *Linnemannia yunnanensis* and *L. bainierella* are closely related. Morphologically, *L. yunnanensis* has larger and differently shaped sporangiospores when compared with *L. bainierella*, and chlamydospores are observed in *L. yunnanensis* but not in *L. bainierella*. *Linnemannia ovalispora* sp. nov. and *L. rugosa* are closely allied. Compared with *L. rugosa*, *L. ovalispora* has differently shaped sporangiospores and chlamydospores. *Mortierella irregularispora* and *M. parvispora* exhibit a high degree of genetic similarity. Compared to *M. parvispora*, *M. irregularispora* has smaller sporangia and larger sporangiospores, and no chlamydospores are observed in *M. irregularispora*.

Soil samples were collected from the humus layer of secondary broad-leaved forests in Yunnan Province and alpine meadows in Xizang Autonomous Region. The Yunnan Province of China is located in the central low-latitude plateau region, with a monsoon climate of the central subtropical humid and cold winter plateau. The Xizang Autonomous Region of China is located on the Qinghai-Tibet Plateau, with a predominantly highland and mountainous climate, further evidence that Mortierellaceae species are widely distributed and cover a wide range of ecosystems (Kuhnert et al. 2012).

Future research should explore the morphological characteristics of Mortierellaceae species, particularly their responses to different growth conditions. Additionally, studying their ecological habits and potential biological activities will contribute to a better understanding of the biology of these new species. Finally, the discovery of these four new species within the Mortierellaceae underscores the need for a more extensive, in-depth investigation of the family. Such studies will shed light on the biodiversity and evolutionary history of the Mortierellaceae family.

### ﻿Supplementary materials

The following supporting information can be downloaded at: The GenBank accession number for the sequence used in this study (Suppl. material 1).

## Supplementary Material

XML Treatment for Linnemannia chlamydospora

XML Treatment for Linnemannia ovalispora

XML Treatment for Linnemannia yunnanensis

XML Treatment for Mortierella irregularispora

## ﻿Additional information

### Use of AI

No use of AI was reported.

### Author ORCIDs

Xin-Yu Ji https://orcid.org/0009-0000-4121-9103

Zi-Ying Ding https://orcid.org/0009-0003-1618-5740

Wen-Xiu Liu https://orcid.org/0009-0002-3283-3704

Fei Li https://orcid.org/0009-0002-7969-6671

Heng Zhao https://orcid.org/0000-0003-2938-5613

Shi Wang https://orcid.org/0000-0002-7376-7638

Xiao-Yong Liu https://orcid.org/0000-0002-8808-010X

### Data availability

The sequences of this study have been submitted to the NCBI database (https://www.ncbi.nlm.nih.gov/, accessed July 15, 2025) with accession numbers were shown in Suppl. material 1.

## Supplementary materials

Supplementary material 1 GenBank accession numbers Data type docx Explanation note GenBank accession numbers of *Linnemannia* and *Mortierella* genus used in this study. The newly discovered species identified in the present study are in bold. Ex-type strains are marked with a star marker “*”. NA stands for “not available”. https://binary.pensoft.net/file/1471537This dataset is made available under the Open Database License (http://opendatacommons.org/licenses/odbl/1.0/). The Open Database License (ODbL) is a license agreement intended to allow users to freely share, modify, and use this Dataset while maintaining this same freedom for others, provided that the original source and author(s) are credited.Xin-Yu Ji, Zi-Ying Ding, Wen-Xiu Liu, Fei Li, Heng Zhao, Shi Wang, Xiao-Yong Liu
