# Supplementary material for: ﻿Unveiling species diversity within Mortierellomycota from China X: Three new species in Linnemannia and one in Mortierella
Source: MycoKeys. 2025 Nov 20;125:245–62. doi: 10.3897/mycokeys.125.168474 (PMC12661329; doi:10.3897/mycokeys.125.168474)
Supplement: Supplementary material 1 — GenBank accession numbers [file mycokeys-125-245-s001.docx]

**Table S1.** GenBank accession numbers of *Linnemannia* genus used in this study.

| **Species** | **Strains** | **ITS** | **LSU** | **SSU** | ***RPB1*** | ***Act*** |
| --- | --- | --- | --- | --- | --- | --- |
| *L. acrotona** | CBS 386.71 | NR_111574 | NA | NA | NA | NA |
| *L. amoeboidea** | CBS 889.72 | NR_111579 | NA | NA | NA | NA |
| *L. bainierella* | Pr1s21 | MT380864 | MZ981756 | NA | NA | NA |
| *L. bainierella* | Pr1s13 | MT380862 | NA | NA | NA | NA |
| *L. bainierella* | Pr1s20 | MT380866 | NA | NA | NA | NA |
| *L. biramosa* | SYFGD6-2 | KP744414 | NA | NA | NA | NA |
| *L. biramosa* | SYFGP2-1 | KP744415 | NA | NA | NA | NA |
| *L. biramosa* | RS5 | PP703041 | NA | NA | NA | NA |
| *L. brevisphora** | CGMCC 3.28577 | PQ844569 | PQ844576 | NA | NA | NA |
| *L. camargensis** | CBS 221.58 | NR_111577 | NA | NA | NA | NA |
| *L. camargensis** | CBS 221.58 | MH857763 | MH869294 | NA | NA | NA |
| ***L. chamydospora**** | **CGMCC 3.28892** | **PV660600** | **PV650841** | **PV826211** | **PV951245** | **PV951247** |
| ***L. chamydospora*** | **XG10460-10-1** | **PV660601** | **PV650842** | **PV826212** | **PV951246** | **PV951248** |
| *L. diaoluoshan** | XG08196-6 | PV125053 | PV125128 | NA | NA | NA |
| *L. elizabethkennyiae** | BRIP 74948a | NR_189983 | OR259051 | OR271910 | NA | NA |
| *L. elongata* | 7 | PQ678932 | NA | NA | NA | NA |
| *L. exigua* | NNIBRFG5521 | ON715840 | ON715849 | NA | NA | NA |
| *L. fatshederae** | CBS 388.71 | NR_182454 | MH871946 | NA | NA | NA |
| *L. fluviae** | EML-YR25716-1 | KX227755 | NA | NA | NA | NA |
| *L. friederikiana** | Pr3s8 | MT308723 | MZ981755 | NA | ON774872 | NA |
| *L. gamsii** | CBS 749.68 | NR_152954 | MH870946 | MH859222 | NA | NA |
| *L. hyaline** | CBS 223.35 | NR_163542 | MH867166 | MH855655 | NA | NA |
| *L. longigemmata** | CBS 653.93 | NR_182440 | NA | NA | NA | NA |
| *L. mannui* | Pr2s5 | MW042230 | MZ981765 | NA | ON774871 | NA |
| *L. nantahalensis** | CBS 610.70 | NR_145300 | NA | NA | NA | NA |
| *L. nimbosa** | HFSF57 | MW042228 | MZ981762 | NA | ON774865 | NA |
| *L. olea** | CGMCC 3.28579 | PQ844573 | PQ844580 | NA | NA | NA |
| ***L. ovalispora**** | **CGMCC 3.28891** | **PV660598** | **PV650839** | **PV826213** | **PV982798** | **PV910484** |
| ***L. ovalispora*** | **XG09524-11-2** | **PV660599** | **PV650940** | **PV826214** | **PV982799** | **PV910485** |
| *L. rhizomorpha* | XG07310-1 | PV563875 | PV563895 | NA | NA | NA |
| *L. rotunda** | CGMCC3.28764 | PV113446 | PV113454 | PV113462 | PV294740 | PV278672 |
| *L. rotunda* | XG08755-7-2 | PV113447 | PV113455 | PV113463 | PV294741 | PV278673 |
| *L. rugosa** | XG10541-6 | PV259388 | PV259380 | NA | NA | NA |
| *L. schmuckeri** | CBS 295.59 | NR_111578 | MH869405 | MH857867 | NA | NA |
| *L. sclerotiella** | CBS 529.68 | NR_145298 | NA | NA | NA | NA |
| *L. scordiella** | HFSF81 | MW042238 | MZ981760 | NA | MZ779209 | NA |
| *L. solitaria** | OAS3 | MT279272 | NA | NA | NA | NA |
| *L. stellaris** | Ks2-4 | MW042232 | MZ981764 | NA | ON774866 | NA |
| *L. tamarindoides** | CGMCC 3.28576 | PQ844568 | PQ844575 | NA | NA | NA |
| ***L. yunnanensis**** | **CGMCC 3.28890** | **PV660596** | **PV650837** | **PV826209** | **PV978096** | **PV910486** |
| ***L. yunnanensis*** | **XG08668-7-2** | **PV660597** | **PV650838** | **PV826210** | **PV978097** | **PV910487** |
| *L. zychae** | CBS 316.52 | NR_111576 | NA | MH857054.1 | NA | NA |
| *Mortierella. longicollis** | CBS 209.32 | HQ630287 | NA | NA | NA | NA |

**Table S2.** GenBank accession numbers of *Mortierella* genus used in this study.

| **Species** | **Strains** | **ITS** | **LSU** | **SSU** | ***RPB1*** | ***Act*** |
| --- | --- | --- | --- | --- | --- | --- |
| *M. acrotona** | CBS 386.71 | JX975921 | HQ667405 | HQ667489 | NA | NA |
| *M. acuta** | CGMCC3.28761 | PV113442 | PV113450 | PV113458 | PV294736 | PV268287 |
| *M. acuta* | XG08182-4-2 | PV113443 | PV113451 | PV113459 | PV294737 | PV268288 |
| *M. alpina** | CBS 210.32 | JX975853 | MH866743 | JQ040258 | JN985287 | NA |
| *M. alpina* | CBS 210.32 | HQ630345 | HQ667421 | HQ667501 | NA | NA |
| *M. amoeboidea** | CBS 889.72 | HQ630346 | HQ667422 | HQ667502 | NA | NA |
| *M. angusta** | CBS 293.61 | HQ630279 | HQ667358 | HQ667443 | NA | NA |
| *M. antarctica** | CBS 609.70 | HQ630347 | HQ667423 | HQ667503 | MN743904 | NA |
| *M. armillariicola** | CBS 914.73 | HQ630282 | HQ667361 | HQ667446 | NA | NA |
| *M. bainieri* | CBS 220.35 | MH855653 | MH867164 | NA | NA | NA |
| *M.basiparvispora** | CBS 517.72 | JX976048 | MH872255 | MH860551 | NA | NA |
| *M. beljakovae** | CBS 123.72 | HQ630352 | HQ667428 | NA | NA | NA |
| *M. biramosa* | CBS 370.95 | JX976094 | HQ667389 | HQ667473 | NA | NA |
| *M. calciphila** | WA 18944 | KT964845 | NA | NA | NA | NA |
| *M. camargensis** | CBS 221.58 | HQ630331 | HQ667408 | HQ667492 | NA | NA |
| *M. capitata* | CBS 110.640 | JX975923 | NA | NA | NA | NA |
| *M. chlamydospora* | CBS 120.34 | HQ630354 | HQ667430 | HQ667508 | NA | NA |
| *M. clonocystis** | CBS 357.76 | HQ630318 | HQ667395 | HQ667479 | NA | NA |
| *M. cogitans** | CBS 879.97 | HQ630281 | HQ667360 | HQ667445 | NA | NA |
| *M. cystojenkinii** | CBS 456.71 | HQ630348 | HQ667424 | HQ667504 | NA | NA |
| *M. dichotoma** | CBS 221.35 | HQ630316 | HQ667393 | HQ667477 | NA | NA |
| *M. echinosphaera** | CBS 575.75 | GU559985 | HQ667431 | NA | NA | NA |
| *M. elongata* | FSU823 | HQ630337 | HQ667413 | HQ667495 | NA | NA |
| *M. elongata* | FSU822 | HQ630336 | HQ667412 | HQ667494 | NA | NA |
| *M. elongatula** | CBS 488.70 | HQ630349 | HQ667425 | HQ667505 | NA | NA |
| *M. epicladia** | CBS 355.76 | HQ630319 | HQ667396 | HQ667480 | NA | NA |
| *M. epigama** | CBS 489.70 | HQ630290 | HQ667367 | HQ667453 | NA | NA |
| *M. exigua** | CBS 655.68 | HQ630329 | HQ667406 | HQ667490 | NA | NA |
| *M. fimbricystis** | CBS 943.70 | GU559986 | NA | NA | NA | NA |
| *M. formicicola* | CBS 109.589 | JX975933 | JX976140 | NA | NA | NA |
| *M. formicae** | WA 49853 | NR_160334 | KY748017 | KY748015 | NA | NA |
| *M. gamsii** | CBS 749.68 | HQ630340 | HQ667416 | NA | NA | NA |
| *M. gamsii* | CBS 551.73 | HQ630341 | HQ667417 | HQ667498 | NA | NA |
| *M. gemmifera** | CBS 134.45 | HQ630293 | HQ667371 | HQ667456 | NA | NA |
| *M. globalpina** | CBS 360.70 | NR_160121 | MH871462 | MH859709 | NA | NA |
| *M.histoplasmatoides** | CBS 321.78 | HQ630309 | HQ667386 | HQ667470 | NA | NA |
| *M. horticola** | CBS 305.52 | HQ630322 | HQ667399 | HQ667483 | NA | NA |
| *M. hypsicladia** | CBS 116.202 | HQ630302 | HQ667379 | NA | NA | NA |
| *M. humilis** | CBS 222.35 | HQ630325 | HQ667401 | HQ667485 | NA | NA |
| *M. humilis* | FSU828 | HQ630326 | HQ667402 | HQ667486 | NA | NA |
| *M. humilis* | CBS 745.68 | HQ630327 | HQ667403 | HQ667487 | NA | NA |
| *M. indohii** | CBS 720.71 | HQ630298 | HQ667377 | HQ667461 | NA | NA |
| *M. indohii* | FSU830 | HQ630299 | EU736318 | EU736291 | NA | NA |
| *M. indohii* | FSU831 | HQ630300 | HQ667438 | HQ667462 | NA | NA |
| ***M. irregularispora**** | **CGMCC 3.28893** | **PV660602** | **PV650843** | **PV739559** | **PV978094** | **PV910488** |
| ***M. irregularispora*** | **XG00435-2-2** | **PV660603** | **PV650844** | **PV739560** | **PV978095** | **PV910489** |
| *M. kuhlmanii** | CBS 157.71 | HQ630294 | HQ667372 | HQ667457 | NA | NA |
| *M. lapis** | OBS3 | MT380877 | MZ981747 | NA | ON774869 | NA |
| *M. lignicola** | CBS 207.37 | HQ630357 | HQ667435 | HQ667511 | NA | NA |
| *M. longicollis** | CBS 209.32 | HQ630287 | HQ667365 | HQ667451 | JN985283 | LN847392 |
| *M. macrocystis* | CBS 314.85 | JX975974 | NA | NA | NA | NA |
| *M. microzygospora** | CBS 880.97 | HQ630317 | HQ667394 | HQ667478 | NA | NA |
| *M. minutissima* | CBS 307.52 | HQ630323 | HQ667400 | HQ667484 | NA | NA |
| *M. multispora** | KUMCC 200005 | MT031921 | MT032146 | NA | NA | NA |
| *M. mutabilis** | CBS 308.52 | HQ630315 | HQ667392 | HQ667476 | NA | NA |
| *M. nantahalensis** | CBS 610.70 | HQ630311 | HQ667388 | HQ667472 | NA | NA |
| *M. oedema** | CGMCC3.28762 | PV113444 | PV113452 | PV113460 | PV294738 | PV278677 |
| *M. oedema* | XG00420-1-2 | PV113445 | PV113453 | PV113461 | PV294739 | PV278678 |
| *M. paraensis* | CBS 547.89 | HQ630353 | HQ667429 | NA | NA | NA |
| *M. parazychae** | CBS 868.71 | HQ630283 | HQ667362 | HQ667447 | NA | NA |
| *M. parvispora* | CBS 311.52 | EU484279 | HQ667373 | HQ667458 | NA | NA |
| *M. polycephala* | FSU696 | HQ630332 | HQ667409 | HQ667493 | NA | NA |
| *M. polycephala* | FSU866 | HQ630333 | HQ667410 | NA | NA | NA |
| *M. polygonia** | CBS 685.71 | HQ630301 | HQ667378 | HQ667463 | NA | NA |
| *M. pulchella* | CBS 312.52 | HQ630351 | HQ667427 | HQ667507 | NA | NA |
| *M. rishikesha** | CBS 652.68 | HQ630308 | HQ667385 | HQ667469 | NA | NA |
| *M. rostafinskii* | CBS 522.70 | HQ630358 | HQ667436 | HQ667512 | NA | NA |
| *M. sarnyensis** | CBS 122.72 | HQ630313 | HQ667390 | HQ667474 | NA | NA |
| *M. schmuckeri** | CBS 295.59 | HQ630338 | HQ667414 | HQ667496 | NA | NA |
| *M. sclerotiella** | CBS 529.68 | HQ630310 | HQ667387 | HQ667471 | NA | NA |
| *M. selenospora** | CBS 811.68 | HQ630343 | HQ667419 | HQ667499 | NA | NA |
| *M. simplex* | CBS 243.82 | JX975870 | NA | NA | NA | NA |
| *M. spinospora* | XG06904-41 | PV563877 | PV563897 | NA | NA | NA |
| *M. strangulate** | CBS 455.67 | HQ630359 | HQ667437 | HQ667513 | NA | NA |
| *M. stylospora** | CBS 211.32 | HQ630280 | HQ667359 | HQ667444 | NA | NA |
| *M. tibetensis** | CGMCC3.28763 | PV113448 | PV113456 | PV113464 | PV415185 | PV278674 |
| *M. tibetensis* | XG00421-2-2 | PV113449 | PV113457 | PV113465 | PV415186 | PV278675 |
| *M. triangularis* | OAS8 | MT380873 | MZ981741 | NA | ON774868 | NA |
| *M. turficola** | CBS 432.76 | HQ630350 | HQ667426 | HQ667506 | NA | NA |
| *M. verrucosa* | CBS 181.73 | NA | MH878485 | NA | NA | NA |
| *M. verticillata* | CBS 346.66 | JN943799 | JN940870 | HQ667481 | JN985284 | NA |
| *M. wolfii* | CBS 209.69 | HQ630303 | HQ667380 | HQ667464 | JN985290 | NA |
| *M. wolfii* | CBS 612.70 | HQ630304 | HQ667381 | HQ667465 | NA | NA |
| *M. wolfii* | CBS 651.93 | HQ630305 | HQ667382 | HQ667466 | JN985289 | NA |
| *M. wuyishanensis** | CBS 370.95 | NR_152953 | NA | NA | NA | NA |
| *M. yunnanensis** | KUMCC 200009 | MT031917 | MT032142 | NA | NA | NA |
| *M. zonata** | CBS 228.35 | HQ630356 | HQ667433 | NA | NA | NA |
| *M. zychae** | CBS 316.52 | HQ630330 | HQ667407 | HQ667491 | NA | NA |
| *Umbelopsis autotrophica** | CBS 310.93 | HQ630285 | HQ667363 | HQ667449 | NA | NA |

**Notes:** The newly discovered species identified in the present study are in bold. Ex-type strains are marked with a star marker "^*^". NA stands for “not available”.
